# X-ray Structures of Precursors of Styrylpyridine-Derivatives Used to Obtain 4-((*E*)-2-(Pyridin-2-yl)vinyl)benzamido-TEMPO: Synthesis and Characterization

**DOI:** 10.3390/molecules20045793

**Published:** 2015-04-02

**Authors:** Guillermo Soriano-Moro, María Judith Percino, Ana Laura Sánchez, Víctor Manuel Chapela, Margarita Cerón, María Eugenia Castro

**Affiliations:** Laboratorio de Polímeros, Centro de Química, Instituto de Ciencias, Benemérita Universidad Autónoma de Puebla (BUAP), Complejo de Ciencias, ICUAP, Edif. 103H, 22 Sur y San Claudio, C.P. 72570 Puebla, Puebla, Mexico

**Keywords:** X-ray structures, single crystal, styrylpyridine derivatives, 4-((*E*)-2-(pyridin-2-yl)vinyl)benzamido-TEMPO, methylpyridines, *p*-terephthaldehyde

## Abstract

The synthesis and characterization of the precursor isomers *trans*-4-(2-(pyridin-2-yl)vinylbenzaldehyde (**I**), *trans*-4-(2-(pyridin-4-yl)vinylbenzaldehyde (**II**), *trans-*4-(2-(pyridin-2-yl)vinylbenzoic acid (**III**) and (*E*)-4-(2-(pydridin-4-yl)vinylbenzoic acid (**IV**) are reported. These compounds were prepared in order to obtain *trans*-4-((*E*)-2-(pyridin-2-yl)vinyl)benzamide-TEMPO (**V**). Compounds **I** and **II** were obtained by using a Knoevenagel reaction in the absence of a condensing agent and solvent. Oxidation of the aldehyde group using the Jones reagent afforded the corresponding acid forms **III** and **IV**. A condensation reaction with 4-amino-TEMPO using oxalyl chloride/DMF/CH_2_Cl_2_ provided the 4-((*E*)-2-(pyridin-2-yl)vinyl)benzamide-TEMPO. Single crystals of compounds **I**, **II** and **III** were obtained and characterized by X-ray diffraction. Compound **I** belongs to space group *P2_1_/c*, *a* = 12.6674(19) Å, *b* = 7.2173(11) Å, *c* = 11.5877(14) Å, *β* = 97.203(13)° and the asymmetric unit was *Z* = 4, whereas compound **II** was in the space group *P*2_1_, with *a* = 3.85728(9) Å, *b* = 10.62375(19) Å, *c* = 12.8625(2) Å, *β* = 91.722 (2)° and the asymmetric unit was *Z* = 2. Compound **III** crystallized as single colorless needle crystals, belonging to the monoclinic system with space group *P*2_1_, with *Z* = 2, with *a* = 3.89359(7) Å, *b* = 17.7014(3) Å, *c* = 8.04530(12) Å, *β* = 94.4030 (16)°. All compounds were completely characterized by IR, ^1^H-NMR, EI-MS and UV-Vis.

## 1. Introduction

Since the first stable radical compound was synthesized in 1961 [1] a series of stable radical structures, such as nitroxyl, phenoxyl, and hydrazyl have been successfully synthesized. These compounds are stable radical compounds that have an electron spin in spite of being organic materials [2]. Nitrogen heterocyclic nitroxides and their diamagnetic derivatives (sterically hindered amine and hydroxylamine) are known as low-molecular-weight multifunctional antioxidants that can participate in one-electron redox processes. Nitroxides have attenuated oxidative damage in various experimental models, including cultured cells [3], brain injury [4], lipid peroxidation in liver microsomes [5], post-ischemic reperfusion injury in isolated organs [6], and exhibit ionizing irradiation damage in rats and mice [7]. Recent investigations have focused on the synthesis of several molecules modified with nitroxides [8,9,10,11]. For example, 2,2,6,6-tetramethylpiperidine 1-oxyl (TEMPO) and its derivatives have been successfully used in modification processes to graft polar species such as –OH or ester groups onto polymers [12]. Also, molecules bearing nitroxides covalently linked to fluorophore have been used as fluorescence spin-label [13,14,15,16] or nuclear-localizing redox probes [17].

In recent years, dyes have been widely used as photoinitiators of free radical polymerization and as fluorescence probes for spectroscopy studies in monitoring specific chemical properties of the medium in which they are incorporated. Therefore, these compounds permit measurements of medium polarity or degree of cure by measuring the changes in the emission intensity or value of the shift in the emission maximum. Also, these dyes have found applications in the development of organic and molecular-based magnetic materials [18]. Currently, there is great interest in applying molecular-based magnetic materials to the development of spin systems with various properties, particularly with respect to photo- and heat-responsive spin systems. The development of novel spin systems has led to the discovery of conjugated organic spin systems which respond to outside stimuli, such as light, heat, pressure and electrons, *etc.* Attempts have been made to develop spin systems with multiple properties [19].

Distyrylbenzenes and related fluorophores have found widespread applications in optical display devices [20,21,22,23,24,25], as nonlinear optical materials [26,27,28], two-photon absorbers [29,30,31], and for sensing purposes [32,33]. The combination of electron-pair donating and electron-pair accepting (EPA) substituents on a π-system can lead to an efficient internal charge transfer, resulting in bathochromic shifts of the electronic spectra, dual fluorescence [34,35,36,37], and efficient two-photon absorption. These properties depend on the length and nature of the π-system and the strength and position of donor and acceptor groups [38]. In addition, the presence of electron withdrawing groups in the π-system, such as replacing benzene rings with electron deficient heterocycles like pyridine [39,40,41,42], has been a successful route to increase the electron affinity. The introduction of a nitrogen atom into the ring noticeably affects the photophysical and photochemical behavior of stilbene. Our work has focused on model compounds with *trans* conformation distyrylbenzene (DSB) linked with pyridine moieties (styrylpyridines) which had been obtained without the need for catalyst and solvent [43,44,45,46,47,48,49,50,51]. We have also conducted studies to understand their effects on the optics and electronic properties from a theoretical perspective [52]. X-ray studies of model compounds showed that although the molecules were planar, they did not exhibit a total delocalization of their electrons throughout the whole molecule, *i.e.*, they did not exhibit complete aromaticity. X-ray results reported that the conformation of the double bond was *trans*. Therefore, using conjugated compounds such as styrylpyridine attached to TEMPO could be widely used to prolong the lifetime of coatings, bulk polymers, thin films, as well applications to label polymers or as a measure of molecular mass by IR and UV spectroscopy.

In this paper, we report the preparation of precursors to obtain low molecular weight styrylpyridine derivatives bearing TEMPO, and their structures obtained by X-ray crystallography analysis, together with their complete characterization. Our goal is to use these compounds as labels of polymers to measure the polymer molecular mass. The precursors are interesting because of the presence of groups such as –COH and –COOH, that allow different absorption properties.

## 2. Results and Discussion

Several approaches for the preparation for methylpyridines with aromatic aldehydes have recently been carried out. Solvent-free methods at temperatures of 120–140 °C were successfully used to synthesize different styrylpyridines and α,β-acrylonitriles as well as intermediates formed in the condensation reaction of methylpyridines and aromatic aldehyde compounds [53,54]. The procedure to obtain the compounds **I** and **II** allowed us to control the reaction of just one aldehyde group from terephthalaldehyde during the condensation reaction with the methyl group that formed the double bond. The behavior of this reaction is attributed to the control of the reaction temperature rather than to the molar ratios of reactants, because when the reaction was carried out at higher temperature, compounds such as 1,4-bis(2,2-diphenylethenyl)benzene or 1,4-bis(4,4-diphenylethenyl)benzene were formed instead of **I** and **II**. Our results also showed that the position of the methyl group on the pyridine ring affected its reactivity. Compounds **III** and **IV** were prepared with Jones oxidation conditions allowing their preparation in a convenient and safe procedure. The modest yield was attributed to the effect of the nitrogen atom in the *ortho* position, affording lower solubility to compound **IV**. In addition, we obtained **III** as the pure acid by recrystallization with CH_3_OH:DMF (80:20).

### 2.1. Characterization

**Figure 1:**
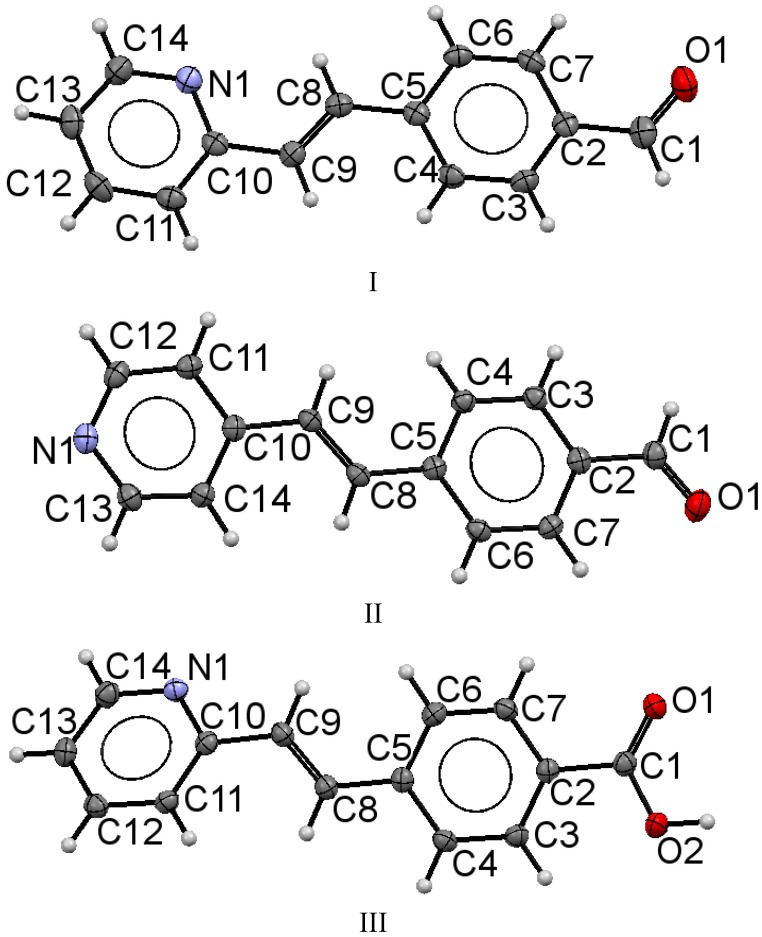
Molecular structures of **I**, **II** and **III**. The displacement ellipsoids are drawn at the 50% probability level and H atoms are shown as small spheres of arbitrary radii.

Characterization from IR, ^1^H-NMR, and EI spectra of **I**–**V** showed characteristic features indicating that the precursors with the isomers structures of *trans*-(*E*)-4-(2-(pyridin-2-yl)vinyl- and *trans*-(*E*)-4-(2-(pyridin-4-yl)vinyl- contained the functional group –COH, –COOH. The ORTEP structures of **I**–**III** including the atomic numbering scheme are shown in Figure 1. Selected crystal data, structure solution and refinement for isomers **I**, **II** and **III** are listed in Table 1. Table 2 gives selected bond lengths with estimated standard deviations also for all three compounds.

**Table 1:** Crystallography Data for **I**, **II** and **III** compounds.

	I	II	III
Empirical formula	C_14_H_11_NO	C_14_H_11_NO	C_14_H_11_NO_2_
Crystal system	monoclinic	monoclinic	monoclinic
Color, Habit	colorless irregular block	yellow, block	Needle, colorless
Formula weight	209.24	209.24	225.24
Space group	*P 2_1_/c*	*P*2_1_	*P 2_1_*
*T* (*K*)	110(2)	100(2)	110(2)
*A* (Å)	12.6674(19)	3.85728(9)	3.89359(7)
*b* (Å)	7.2173(11)	10.62375(19)	17.7014(3)
*c* (Å)	11.5877(14)	12.8625(2)	8.04530(12)
α (°)	90.00	90.00	90.00
β (°)	97.203(13)	91.722(2)	94.4030(16)
γ (°)	90.00	90.00	90.00
*V* (Å^3^)	1051.0(3)	526.852(18)	552.862(16)
*Z*	4	2	2
Dc(g cm^−3^)	1.322	1.319	1.353
*F* (000)	592	220	440
μ (mm^−1^)	0.663	0.662	0.740
λ (Å)	1.5418	1.5418	1.5418
Crystal size (mm^3^)	0.25 × 0.10 × 0.03	0.39 × 0.28 × 0.22	0.24 × 0.09 × 0.06
2θmax (°)	143.8	143.8	143.8
No of reflections	6697	6136	7517
N° of unique reflections, I > 2 σ(I)	1933	2037	2165
R_1_ (I > 2 σ(I)), R_1_ (all)	6.08, 8.29	3.03, 3.05	2.75, 2.79
wR_2_ (I > 2 σ(I)), wR_2_ (all)	17.16, 18.39	8.63, 8.65	7.43, 7.48
goodness-of-fit	1.169	1.085	1.054
Largest diff peak and hole (e Å^−3^)	0.22 and −0.27	0.21 and −0.21	0.21 and −0.16

**Table 2:** Bond lengths (Å) for compounds **I**, **II** and **III**.

	I	II	III
C(1)-O(1)	1.212(4)	1.212(2)	1.219(2)
C(1)-C(2)	1.470(4)	1.477(2)	1.493(2)
C(2)-C(3)	1.391(4)	1.392(2)	1.392(2)
C(2)-C(7)	1.398(4)	1.394(2)	1.398(3)
C(4)-C(5)	1.400(4)	1.402(2)	1.399(3)
C(5)-C(6)	1.406(4)	1.403(2)	1.401(2)
C(5)-C(8)	1.462(4)	1.470(2)	1.466(2)
C(6)-C(7)	1.376(4)	1.386(2)	1.383(3)
C(8)-C(9)	1.331(4)	1.331(2)	1.335(3)
C(9)-C(10)	1.469(4)	1.469(2)	1.470(2)
C(10)-C(11)	1.399(4)	1.393(2)	1.397(3)
C(11)-C(12)	1.382(5)	1.391(2)	1.381(3)
C(13)-C(14)	1.395(5)	1.388(2)	1.386(3)
C(12)-C(13)	1.370(5)		1.386(3)
C(10)-N(1)	1.351(4)		1.352(2)
C(14)-N(1)	1.330(4)		1.335(2)
C(1)-O(2)			1.317(2)
C(10)-C(14)		1.397(2)	
C(12)-N(1)		1.338(2)	
C(13)-N(1)		1.343(2)	

**Figure 2:**
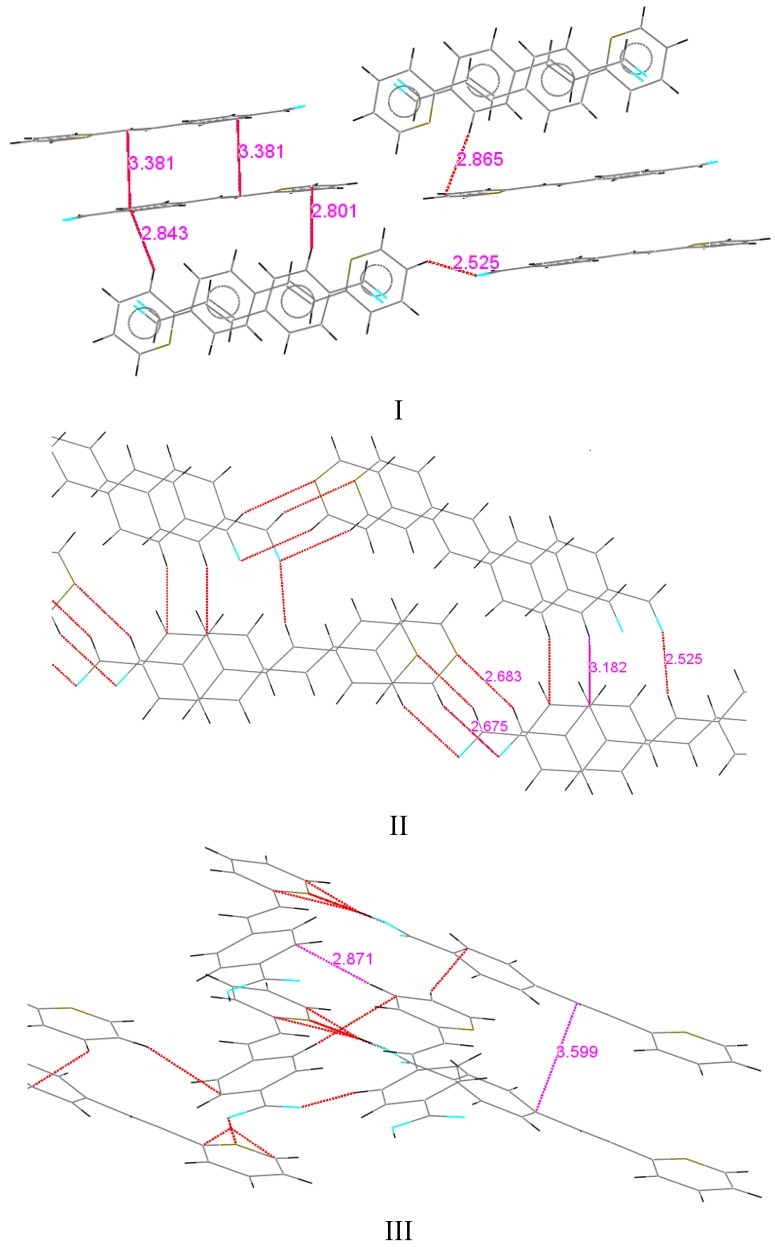
Molecular crystal packing of **I**, **II** and **III** short contacts (dashed red lines).

The molecular structures for compounds **I** and **II** were ordered. The 4-phenylcarbaldehyde moiety is located *trans* to the pyridine ring in the molecule in relation to the double bond. The bond lengths for compound **I** were C(10)-C(9) [1.469(4) Å], C(9)-C(8) [1.331(4) Å], C(8)-C(5) [1.462(4) Å], (Table 2) while the corresponding bonds lengths for the compound **II** were 1.469(2) Å 1.331(2) Å, 1.470(2) Å. These values indicated that the compounds contained a double bond in conjugation with an aromatic ring, (C*sp*^2^=C*sp*^2^; *trans* of 1.32 Å conjugation; C*sp^2^*-C*ar* (C=C-C*ar* conjugated at 1.47 Å) as well as a delocalization of the π electrons C*ar*
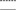
C*ar* in the phenyl ring of 1.387 Å, in the two six-membered rings through the C(8)-C(9) bond. Similar distances were observed for the 2-styrylpyridine derivatives and were slightly different than the reported values [55]. Bond lengths C(1)-O(1) for **I** and **II** were of the equal value, 1.212(4) Å for aldehyde (C*sp^2^*=O in Car-C=O of 1.221 Å) [55]. Also, the results showed that structures **I** and **II** were almost planar molecules, with the pyridyl ring and 4-phenylcarbaldehyde group being coplanar with the double bond (Table 3 and Table 4). For **I**, the torsion angles between the atoms C(8)-C(9)-C(10)-N(1) were 0.4(5)°, C(4)-C(5)-C(8)-C(9) of 1.0(5)° and between O(1)-C(1)-C(2)-C(3) were −172.1(3)°. Compound **II** showed torsion angles between C(8)-C(9)-C(10)-C(14) of 1.2(3)°, C(4)-C(5)-C(8)-C(9) of −4.6(2)°, and between O(1)-C(1)-C(2)-C(3) of 179.57(16)°. The partial π character of the C(9)-C(8), C(10)-C(9) and C(8)-C(5) bonds (Table 2) helps to explain the aromatic planar nature of the molecules. The difference between both compounds is in the molecular crystal packing (Figure 2). For **I**, the molecular packing does not present regular hydrogen bonding between molecules. However, the packing motif showed a non-classical herringbone packing with a weak π-π overlap between neighboring molecules. For compound **I**, the distances between the co-facial π-π overlap interaction C/π…C/π was 3.381 Å, between CH(py)**^…^**C(Ar) (2.843 Å) and C(Ar)**^…^**CH(py) (2.865 Å). Within each stack, however, the molecules are translated (slipped) along the short axis, thus minimizing the π-overlapping between them. A similar interaction arises for compound **II** between CH(COH)**^…^**N(Py) of 2.683 Å, and C(CO)**^…^**CH(Py) 2.615 Å, but **II** did not present short contact interactions such as CH/π∙∙∙∙–CH/π.

**Table 3:** Bond angles (°) for compounds **I**, **II** and **III**.

	I	II	III
O(1)-C(1)-C(2)	124.5(3)	124.68(16)	122.50(17)
C(3)-C(2)-C(1)	119.0(3)	118.97(15)	121.85(17)
C(7)-C(2)-C(1)	122.1(3)	121.11(15)	119.15(16)
C(4)-C(5)-C(8)	122.8(3)	122.25(14)	119.27(16)
C(6)-C(5)-C(8)	119.4(3)	118.76(13)	122.46(16)
C(9)-C(8)-C(5)	126.4(3)	126.24(14)	125.65(17)
C(8)-C(9)-C(10)	124.7(3)	125.54(14)	124.62(17)
C(11)-C(10)-C(9)	119.9(3)	119.68(14)	123.49(16)
N(1)-C(14)-C(13)	124.0(3)	124.18(15)	122.86(19)
N(1)-C(10)-C(11)	122.0(3)		120.93(17)
N(1)-C(10)-C(9)	118.1(3)		115.58(16)
C(13)-C(12)-C(11)	119.4(3)		119.76(18)
C(12)-C(13)-C(14)	118.1(3)		118.04(18)
C(14)-N(1)-C(10)	117.5(3)		119.27(16)
C(11)-C(10)-C(14)		116.77(14)	
C(14)-C(10)-C(9)		123.54(15)	
N(1)-C(12)-C(11)		124.19(16)	
C(13)-C(14)-C(10)		119.44(15)	
C(12)-N(1)-C(13)		115.91(14)	
C(1)-O(2)-H(2)			114.4(18)
O(2)-C(1)-C(2)			113.39(16)
O(1)-C(1)-O(2)			124.11(17)

**Table 4:** Torsion angles (°) for compounds **I**, **II** and **III**.

	I	II	III
O(1)-C(1)-C(2)-C(3)	−172.1(3)	179.57(16)	170.38(19)
O(1)-C(1)-C(2)-C(7)	7.5(5)	−1.9(3)	−8.9(3)
C(4)-C(5)-C(8)-C(9)	1.0(5)	−4.6(2)	−165.4(2)
C(6)-C(5)-C(8)-C(9)	179.5(3)	175.87(15)	15.8(3)
C(5)-C(8)-C(9)-C(10)	178.9(3)	178.74(15)	−179.94(17)
C(8)-C(9)-C(10)-C(11)	−178.5(3)	−178.21(15)	19.0(3)
C(8)-C(9)-C(10)-N(1)	0.4(5)		−161.1(2)
N(1)-C(10)-C(11)-C(12)	−0.8(5)	0.0(3)	1.9(3)
C(9)-C(10)-C(11)-C(12)	178.1(3)	178.96(14)	−178.31(19)
C(9)-C(10)-N(1)-C(14)	−178.8(3)		178.04(16)
C(10)-C(11)-C(12)-C(13)	0.7(5)		0.0(3)
C(11)-C(12)-C(13)-C(14)	−0.1(5)		−1.6(3)
C(12)-C(13)-C(14)-N(1)	−0.6(5)		1.4(3)
C(13)-C(14)-N(1)-C(10)	0.6(5)	0.1(2)	0.5(3)
C(11)-C(10)-N(1)-C(14)	0.1(4)		−2.1(3)
C(8)-C(9)-C(10)-C(14)		1.2(3)	
C(14)-C(10)-C(11)-C(12)		−0.5(2)	
C(11)-C(10)-C(14)-C(13)		0.4(2)	
C(9)-C(10)-C(14)-C(13)		−178.99(15)	
C(11)-C(12)-N(1)-C(13)		0.5(2)	
C(14)-C(13)-N(1)-C(12)		−0.6(2)	
O(2)-C(1)-C(2)-C(3)			−9.3(3)
O(2)-C(1)-C(2)-C(7)			171.38(17)

In contrast, for **III**, the structure was comprised of three groups, phenyl, pyridyl and a double bond; the –COOH group was not completely coplanar due most likely to the intermolecular H-bonding between OH group of one molecule N and nitrogen atoms of a symmetry-related pyridine ring [O(2)-H(2)^…^N(1), 1.669 Å, O(2)-H(2), 0.97(3) Å, O(1)^…^N(1) 2.642 Å, O(2)-H(2)^…^N(1) 178.29°], symmetry code: 2−x, −1/2+y, 1−z. Figure 2 illustrates that in the molecular structure, the styryl-moiety is moved out the plane that contains the pyridine ring. The torsion angle (Table 4), between C(6)-C(5)-C(8)-C(9) is 15.8(3)° and between C(4)-C(5)-C(8)-C(9) is −165.4(2)°. With respect to the pyridine ring, the plane was moved out with torsion angles between C(8)-C(9)-C(10)-N(1) of −161.1(2)°, between C(11)-C(10)-C(9)-C(8) of 19.0(3)°, and between C(15)-C(16)-C(18)-C(19) of −15.7(2)°.

**Scheme 1:**
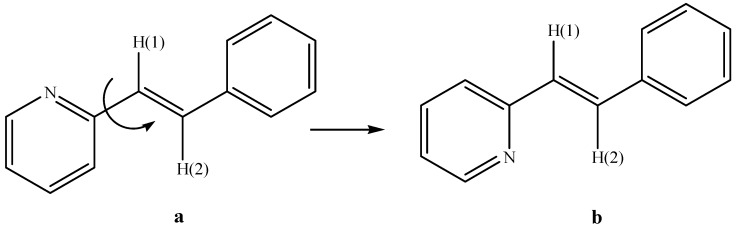
Rotamers where the pyridine group could be in *syn* (**a**) or *anti* (**b**) configuration for 2-styrylpyridine.

Figure 1 and Figure 2 show that compound **III** displayed a rotation in the opposite direction to that of compounds **I** and **II**. This was an interesting outcome because even though the double bond exhibited a *trans* configuration, its position showed a different arrangement compared to several previously reported styrylpyridines [43,44,45,46,47,48,49,50,51]. According to a recently calculated report [52] of the energy for the conformation of 2-styrylpyridine, the nitrogen atom of the pyridine could rotate around the bond between the double bond and the pyridine ring to diminish the steric effects of the proton of the ring with the protons of double bond H(1) or H(2) (Scheme 1). In the calculated results, the energy corresponding to the *syn* form (a) is 5.561 KJ mol^−1^ and for *trans* form (b) is 4.536 KJ mol^−1^, indicating that rotamer (b) should be more stable than (a), by almost 3 orders of magnitude order at the theory level. In contrast to the X-ray crystallography of **III**, in its molecular structure, the proton H(1) is located *syn* to the nitrogen atom even in the presence of a free pair of electrons that should contribute to destabilization of the molecule, according to different reports. This configuration is an indication of the possible presence of different rotamers. However, the molecular structure of **III** was obtained with proton H(1) located in a *syn* configuration. The molecular packing also showed weaker intermolecular interactions in form of face to face and side by side (π-π) molecular stacking.

### 2.2. Characterization of trans-4-((E)-2-(Pyridin-2-yl)vinyl)benzamido-TEMPO (**V**)

**Figure 3:**
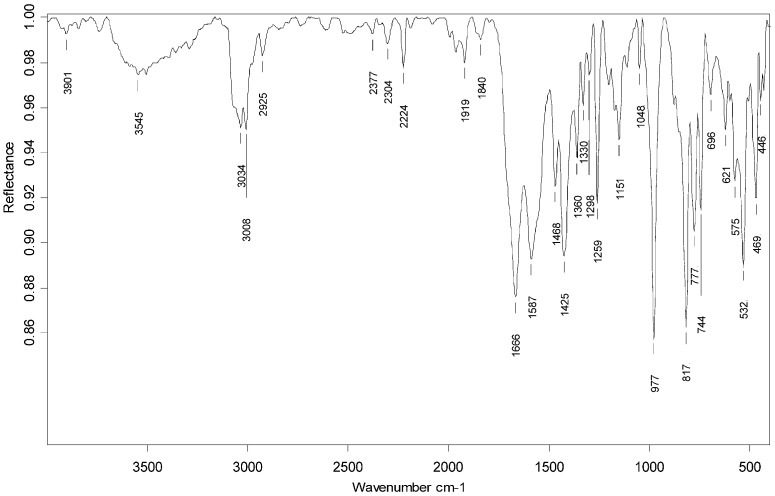
IR spectrum corresponding to *trans-*4-((*E*)-2-(pyridin-2-yl)vinyl)benzamido-TEMPO (**V**).

Characterization by IR of *trans-*4-((*E*)-2-(pyridin-2-yl)vinyl)benzamido-TEMPO (**V**) (Figure 3) showed characteristic bands at 1661 cm^−1^ and 1587 cm^−1^ which were assigned to the ν(C=O) and ν(C-N) corresponding to the amide group (−C(O)NH) present in the compound. Also, the vibration corresponding to ν(N-O^•^) from nitroxyl group is observed at 1360 cm^−1^, which is in agreement with the experimental and calculations reports [56,57,58]. In addition, bands at 3545 cm^−1^, 3008 cm^−1^, 2925 cm^−1^ and 977 cm^−1^ which correspond to ν(N-H), ν(C-H, aromatic), ν(CH_2_) and δ(=C-H, *trans*) vibration are observed. Also, at 1259 cm^−1^ there is a weak band corresponding to the interaction between N-H bending and C-N stretching of the amide group. This spectral evidence gave information about the formation of **V**. Currently, investigations to acquire the ^1^H-NMR spectrum of the corresponding hydroxylamine analogue are underway. The outcome of these studies should give more evidence regarding the conformation of **V**.

### 2.3. One Photon Absorption Characterization

**Figure 4:**
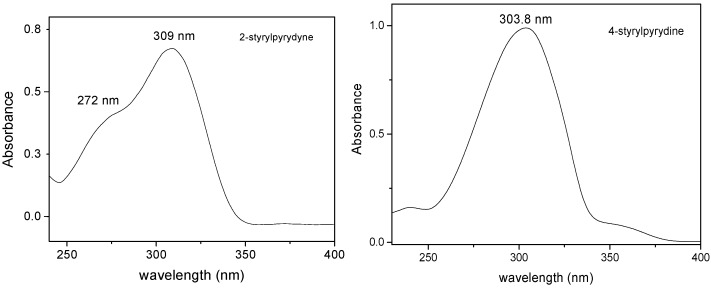
Absorption spectra of 2-styrylpyridine and 4-styrylpyridine in methanol.

**Figure 5:**
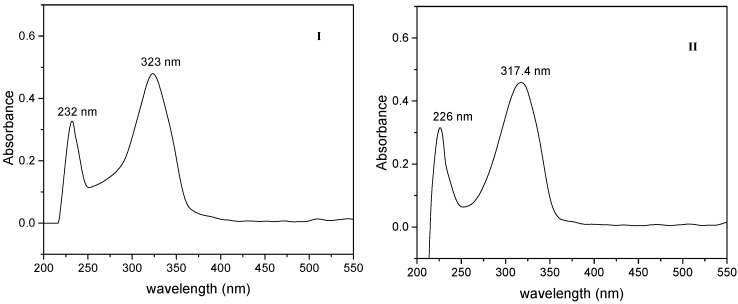
Absorption spectra of (**I**) and (**II**).

**Figure 6:**
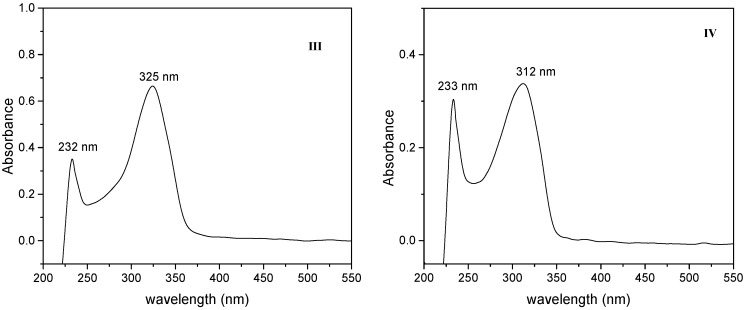
Absorption spectra of the (**III**) and (**IV**) respectively.

**Figure 7:**
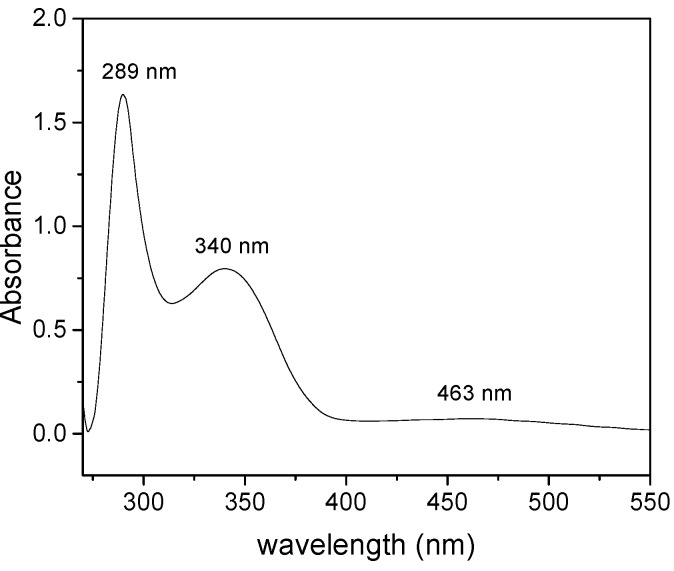
Absorption spectrum of (**V**).

Absorption spectra of the 2-styrylpyridine and 4-styrylpyridine (Figure 4) were recorded in methanol in allow comparison with the compounds **I**, **II**, **III** and **IV** and to evaluate the effect of the −CHO and −COOH substituents (Figure 5 and Figure 6). An insignificant red shift in the absorption wavelength occurred due to presence of −COH for both isomers. The 2-styrylpyridine compound exhibited one band at 309 nm corresponding to the π→π* transition characteristic for a double bond in the *trans* position; this result agreed well with previous reports [45,50,52]. 4-Styrylpyridine exhibited an absorption band at 303 nm corresponding to the π→π* transition due to the double bond, which is in agreement with theoretical reports [59,60]. The spectra for both isomers **I** and **II** displayed a significant red shift as compared with the corresponding 2- and 4-styrylpyridine compounds (Figure 5) due to the presence of the −COH in both isomers. Compound **I** exhibited a stronger red shift than did **II**. However, for compounds **III** and **IV** in the acid form, the −COOH group had little effect on the absorption wavelength position (Figure 6). Compounds **III**–**IV** showed an absorption at 232 nm, which could be assigned to the transition of the n→π* of the electron-withdrawing −COOH. This red shift was related to intermolecular hydrogen bonding interactions between two adjacent groups. The spectrum of compound **V** (Figure 7) was completely different, but the band at λ_max_ 289 nm could attribute to the chromophore N-O present in the structure [61].

## 3. Experimental Section

### 3.1. Materials and Instrumentation

Methylpyridines, *p-*terephthalaldehyde, NH_2_-TEMPO, oxalyl chloride, DMF, and CH_2_Cl_2_, were acquired from Aldrich Chemical Co. (Toluca, México) and were purified before use. The Jones reagent was prepared during the oxidation reaction. Melting points were measured with an SEV (0–300 °C) apparatus and were reported as uncorrected values. IR spectra of the products were recorded on a Vertex (model 70, Bruker Optics, Ettlingen, Germany) 750 FT-IR spectrophotometer by attenuated total reflectance (ATR). ^1^H-NMR and ^13^C-NMR spectra were obtained in CDCl_3_ and DMSO-*d*_6_, on a Varian 400 MHz NMR spectrometer (Varian NMR, Walnut Creek, CA, USA). The electron ionization (EI) spectra were acquired on a JeolMStation 700-D mass spectrometer (Jeol USA, Peabody, MA, USA). UV-Vis spectra were acquired with a Spectrometer SD2000 (Ocean Optics, Dunedin, FL, USA) equipped with a pulse Xenon light source PX-2 (Ocean Optics). The solvent used for measurements in solution was methanol of spectroscopic grade and was preliminarily checked for the absence of absorbing impurities within the scanned spectral ranges.

### 3.2. Synthesis and Characterization of Precursors **I**–**IV**

**Scheme 2:**
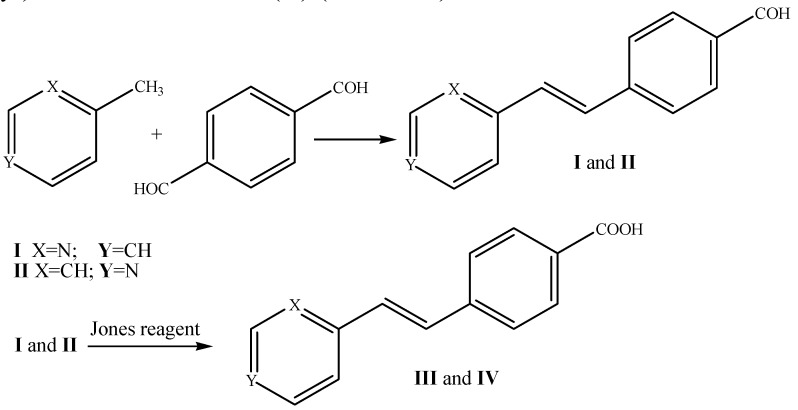
Syntheses of *trans*-4-(2-(pyridin-2-yl)vinylbenzaldehyde (**I**), *trans*-4-(2-(pyridin-4-yl)vinylbenzaldehyde (**II**), *trans-*4-(2-(pydridin-2-yl)vinylbenzoic acid (**III**), (*E*)-4-(2-(pydridin-4-yl)vinylbenzoic acid (**IV**).

**Scheme 3:**
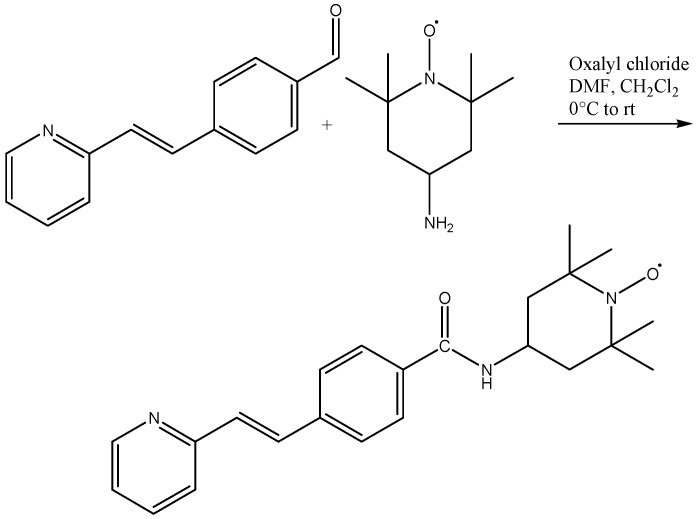
Synthesis of *trans-*4-((*E*)-2-(pyridin-2-yl)vinyl)benzamido-TEMPO (**V**).

The precursors *trans*-4[-(2-(pydridin-2-yl)vinyl)]benzaldehyde (**I**) and *trans*-4[-(2-(pyridin-4-yl)vinyl]-benzaldehyde (**II**) were prepared as follows. The basic structures of (**I**) and (**II**) were modified by oxidation of the –COH group to a carboxylic acid **(III** and **IV**) (Scheme 2). Then, *trans*-(*E*)-4-(2-(pydridin-2-yl)vinylbenzoic acid (**III**) was condensed with amino-TEMPO to obtain the *trans*-4-((*E*)-2-(pyridin-2-yl)vinyl)benzamido-TEMPO (**V**) (Scheme 3).

Compounds **I** and **II** were obtained by slight modifications of the reported methodology [43]. Terephthaldehyde (2.35 g; 25.3 mmol) with 2-methylpyridine for **I** and 4-methylpyridine for **II** (2.5 mL; 25.3 mmol) were refluxed in the absence of a condensing agent at 120 °C for 50–60 h. In both cases the reaction mixtures were oily and had brown or red-brown color. The mixture was treated with a solution of 2 N NaOH (643 mL) to precipitate a solid. The products were purified by recrystallization with cyclohexane and characterized by IR, ^1^H-NMR, EI and single crystal X-ray crystallography. The yields were 18.81% (**I)** and 20.12% (**II)**. The melting point was 80–81 °C and 109–111 °C for (**I**) and (**II**), respectively.

(**I**) IR(KBr), $\tilde{\nu }$*/*cm^−1^: 1695 (sharp, νC=O), 1599 (w, νC=N Py), 1600 (broad, νC=C Ar), 974 (s, δC-H CHR_1_=CHR_2_ in *trans*). ^1^H-NMR (500 MHz, CD_3_Cl) δ (ppm): 10.03 (s, 1H), 8.67–8.66 (dd, 1H, *J*_H-H_ = 5.5; 4.5; 0.5), 7.93–7.91 (dd, 2H, *J*_H-H_ = 8; 6.5; 1.5), 7.76–7.72 (m, 4H, *J*_H-H_ = 8.5; 6; 5.5; 5.0), 7.46–7.44 (d, 1H, *J*_H-H_ = 8), 7.35–7.32 (d, 1H, *J*_H-H_ = 16), 7.25–7.21 (dd, 1H, *J*_H-H_ = 16). EI (*m/z*, %): molecular ion 209 [M^+^, 25], 208 (92), 180 (15), 178 (6), 133 (100), 105 (52), 77 (50).

(**II**) IR (KBr): $\tilde{\nu }$*/*cm^−1^*:*1688 (sharp, νC=O), 1567 (w, νC=N, Py), 1598 (broad, νC=C, Ar), 979 (s, δC-H CHR_1_=CHR_2_ in *trans*). ^1^H-NMR (500 MHz), (CD_3_Cl): δ (ppm): 10.03 (s, 1H), 8.64–8.63 (d, 2H, *J*_H-H_ = 6), 7.93–7.91 (dd, 2H, *J*_H-H_ = 8.5), 7.37–7.34 (d, 1H, *J*_H-H_ = 16), 7.19–7.16 (d, 1H, *J*_H-H_ = 16), 7.71–7.7 (d, 2H, *J*_H-H_ = 8.5), 7.42–7.40 (dd, 2H, *J*_H-H_ = 8; 5). EI (*m/z*, %): 209 [M^+^, 100], 208 (77), 180 (96), 178 (10), 104 (7), 76 (15).

#### 3.2.1. Synthesis and Characterization of *trans*-(*E*)-4-(2-(Pydridin-2-yl)vinylbenzoic acid and *trans*-(*E*)-4-(4-(pydridin-4-yl)vinylbenzoic acid (**III** and **IV**)

For both compounds, the oxidation reaction used the Jones reagent, which was prepared with 0.66 g of CrO_3_ (6.6 mmol) dissolved in H_2_O. To the solution, an adequate quantity of H_2_SO_4_ was added until a red precipitate formed. An acetone solution with 1.38 g (6.6 mmol) **I** or **II** was prepared at room temperature and it was added to the solution of Jones reagent dissolved in small quantity of water. The reaction mixture was refluxed for about 24 h. After the mixture was treated with NaHCO_3_/H_2_O to reach pH = 7, a yellow precipitate (**III** or **IV**) formed. The solid **III** was purified by recrystallization with a solvents mixture of H_2_O/acetone (80:20), whereas **IV** was recrystallized with DMF:CH_3_OH (20:80). Finally, the compounds were treated with heated hexane to eliminate any remains of **I** and **II**. The products **III** and **IV** were characterized by IR, ^1^H-, ^13^C-NMR and EI. Compound **III** was further characterized by single crystal X-ray crystallography. The yields were 39.40% for **III** and 11.27% for **IV**; the melting point of **III** was of 217–220 °C while **IV** did not present a melting point >300 °C.

(**III**) IR (KBr): $\tilde{\nu }$_/_cm^−1^: 1692 (s and broad, νC=O), 1601 (w, νC=C, Ar), 1569 (νC=N, Py), 1281 (s, wide, νC-O), 964 (s, δC-H CHR_1_=CHR_2_ in *trans*). ^1^H-NMR (500 MHz), (DMSO-*d_6_*): δ (ppm): 12.97 (wide signal, 1H) 8.61–8.60 (dd, 1H, *J*_H-H_ = 5.5, 4.5, 3.5), 7.97–7.95(dd, 2H, *J*_H-H_ = 8), 7.84–7.58 (m, 4H, *J*_H-H_ = 16, 8, 6, 5.5), 7.60–7.58 (dd, 1H, *J*_H-H_ =8), 7.48–7.45 (dd, 1H, *J*_H-H_ = 16), 7.31–7.28 (m, 1H, *J*_H-H_ = 5.5). ^13^C (500MHz), (DMSO-*d_6_*) δ (ppm): 167.73 (***C***OOH), 155.16 (Ar), 150.29 (py), 141.35 (py), 137.61 (Ar), 131.55 (Ar), 131.15 (***C***H=CH), 130.8 (Ar), 130.49 (Ar), 127.76 (CH=***C***H), 123.62 (py), 123.52 (py). EI (*m/z*, %): 225 [M^+^, 22], 224 (100), 178 (7), 87 (15).

(**IV**) IR (KBr): $\tilde{\nu }$*/*cm^−1^: 2435 (s, wide, COH**^…^**OC), 1699 (s and broad, νC=O), 1604 (w, νC=C, Ar,), 1567 (w, νC=N, Py), 1286(s, wide, νC-O), 959 (s, δC-H CHR_1_=CHR_2_ in *trans*). ^1^H-NMR (500 MHz), (CD_3_)_2_SO), δ (ppm): 8.59–8.58 (dd, 2H, *J*_H-H_ = 5.5), 7.98–7.96 (d, 2H, *J*_H-H_ = 8.5), 7.79–7.77 (dd, 2H, *J*_H-H_ = 8), 7.65–7.60 (m, 3H, *J*_H-H_ = 17.5, 6), 7.43–7.39 (dd, 1H, *J*_H-H_ = 16.5). ^13^C (DMSO-*d_6_*), δ (ppm): 167.49 (***C***OOH), 150.6 (py), 144.31 (py), 140.85 (Ar), 132.42 (Ar), 131.0 (***C***H=CH), 130.29 (Ar), 128.90 (Ar), 127.57 (CH=**C**H), 121.57 (py). EI (*m/z*, (%): 225 [M^+^, 18], 206 (90), 180 (60), 178 (20), 152 (41), 102 (7), 76 (10).

#### 3.2.2. Condensation Reaction to Obtain the trans-4-((E)-2-(Pyridin-2-yl)vinyl)benzamido-TEMPO (**V**)

To synthesize **V**, carboxylic acid **III** was reacted with 4-NH_2_-TEMPO using oxalyl chloride, DMF and CH_2_Cl_2_ [62] (Scheme 3). To a three-necked flask was added 0.22 g (1 mmol) of **III** dissolved in 5 mL of DMF at 4 °C. The mixture was reacted for 30 min with stirring and then oxalyl chloride (0.22 mL, 2 mmol) was added, and the temperature was increased to room temperature. 4-Amino TEMPO (NH_2_-TEMPO) (0.170 g, 1 mmol) dissolved in DMF was then added. The mixture was stirred for 48 h at 90 °C. During this time the mixture changed from light yellow to orange and finally to orange-pink. After the reaction time, the mixture was treated with acetone/H_2_O (2:1) to precipitate an orange solid, which was isolated by vacuum-filtration. The solid was purified by recrystallization with a mixture of EtOH/ethyl acetate (1:1); yield was 35% with m.p. of 140–142 °C.

### 3.3. Crystallization of Compounds **I**, **II** and **III**

Crystals of **I** were obtained with 55 mg of **I** dissolved in 3 mL of a mixture of solvents THF/hexane (80:20) and kept at room temperature. After 24 h, colorless crystals were formed.

Crystals of **II** were obtained with 55 mg of compound **II** dissolved in 5 mL of EtOH/cyclohexane (80:20). The solution was kept at room temperature and allowed to slowly evaporate and after 2 days, crystals were formed.

Crystals of **III** were obtained with 4 mg of compound **III** dissolved in 5 mL of MeOH/DMF (80:20) at high temperature. The solution was kept at 4 °C and allowed to slowly evaporate. After 8 days, crystals were obtained.

### 3.4. X-ray Crystallography

All reflection intensities of **I** and **III** were measured at 110(2) K and for **II** at 100(2) K using a SuperNova diffractometer (equipped with Atlas detector) with Cu *K*α radiation (λ = 1.54178 Å). The program CrysAlisPro (Version 1.171.36.32 2013, Agilent Technologies, Santa Clara, CA, USA) was used to refine the cell dimensions and for data reduction. The structures were solved with the program SHELXS-2013 and were refined on *F^2^* with SHELXL-2013 [63]. Empirical absorption correction using spherical harmonics implemented in the SCALE3 ABSPACK scaling algorithm was applied using CrysAlisPro (Version 1.171.36.32). The temperature of the data collection was controlled using the system Cryojet (manufactured by Oxford Instruments, Abingdon, Oxford, UK). The H atoms were placed at calculated positions using the instructions AFIX 43 with isotropic displacement parameters having values 1.2 times *U*eq of the attached C atoms. The H atom attached to O_2_ was found from the difference Fourier map, and its coordinates were refined freely.

## 4. Conclusions

We have synthesized precursors of styrylpyridine in good yield and they were well characterized. The crystallography data gave evidence that the molecular structure of the double bond was in the *trans* conformation, as well as that one of the phenyl rings in **III** was twisted appreciably and featured an intermolecular hydrogen bond with one of the neighboring complexes to form a one-dimensional or two-dimensional network. These intermolecular interactions would induce cooperative effects, leading to good conjugation properties. Finally, this methodology has developed a general method to functionalize the synthesized precursors **III**–**VI** with NH_2_-TEMPO.

## Supplementary Materials

Crystallographic data (excluding structure factors) reported in this paper have been deposited with the Cambridge Crystallographic Data Centre as supplementary publication no. CCDC 1054889, 1054890, 1054891. Copies of available material can be obtained, free of charge, on application to the CCDC, 12 Union Road. Cambridge CB2 IEZ, UK, fax: +44-(0)1223-336033 or e-mail: deposit@ccdc.cam.ac.uk.
